# Supplementary material for: Nuclear quantum effects in molecular liquids across chemical space
Source: Nat Commun. 2025 Jul 1;16:5786. doi: 10.1038/s41467-025-60850-x (PMC12219319; doi:10.1038/s41467-025-60850-x)
Supplement: Supplementary file 1 — Supplementary information [file 41467_2025_60850_MOESM1_ESM.pdf]

Supplementary Information for

Nuclear Quantum Effects in Molecular Liquids Across Chemical Space

Baris E. Ugur and Michael A. Webb*

Department of Chemical and Biological Engineering, Princeton University, Princeton, NJ 08544, USA

* Corresponding author. Email: mawebb@princeton.edu

Contents:

Suppl. Note 1. Chemical Structures of Studied Molecules

Suppl. Note 2. Comparison of NQEs on Molar Volume and Density

Suppl. Note 3. Effect of Anharmonicity on Δv_m

Suppl. Note 4. Analysis NQEs Across Various Properties

Suppl. Note 5. Extended SHAP Analysis

Suppl. Note 6. Impact of Molecular Chemistry on Δv_m

Suppl. Note 7. Distribution of T_{v_m} Across Studied Systems

Suppl. Note 8. Extended Analysis of n_H and α_P

Suppl. Note 9. Impact of Various Hydrogen-Bonding Groups on Δv_m

Suppl. Note 10. Effects of Molecular Branching on System Characteristics

Suppl. Note 11. Benchmarking of TAFFI Force Field with Experiment

Suppl. Note 12. NVE Energy Conservation in PIMD Simulations

Suppl. Note 13. Convergence of Density with Ring-Polymer Bead Count

Suppl. Note 14. Impact of Force Field on Δv_m

Suppl. Note 1. Chemical Structures of Studied Molecules

The diverse range of studied chemistries and molecular structures are visualized below.

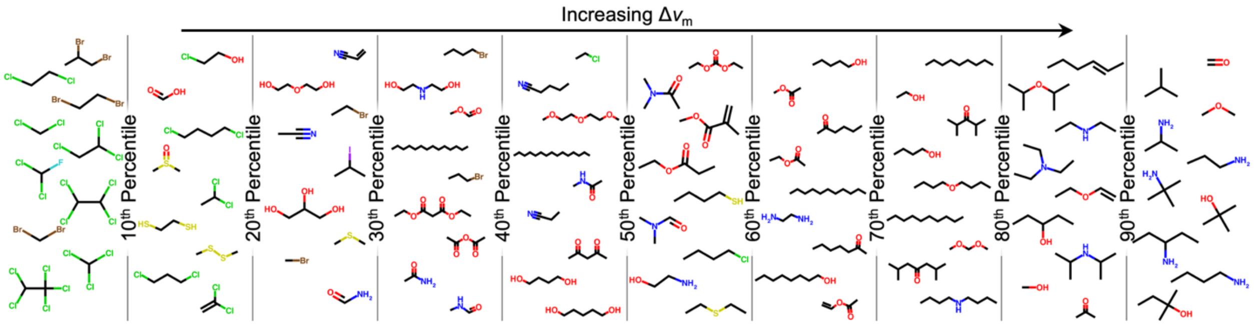

Supplementary Figure 1: Chemical structure drawings of every simulated system. Molecules are categorized into 10 percentile bins based on their Δv_m. Within each bin, higher vertical order also denotes higher Δv_m. Source data are provided as a Source Data file.

Suppl. Note 2. Comparison of NQEs on Molar Volume and Density

To evaluate how nuclear quantum effects on molar volume may connect to effects on density, we compare their magnitudes across the 92 studied molecules.

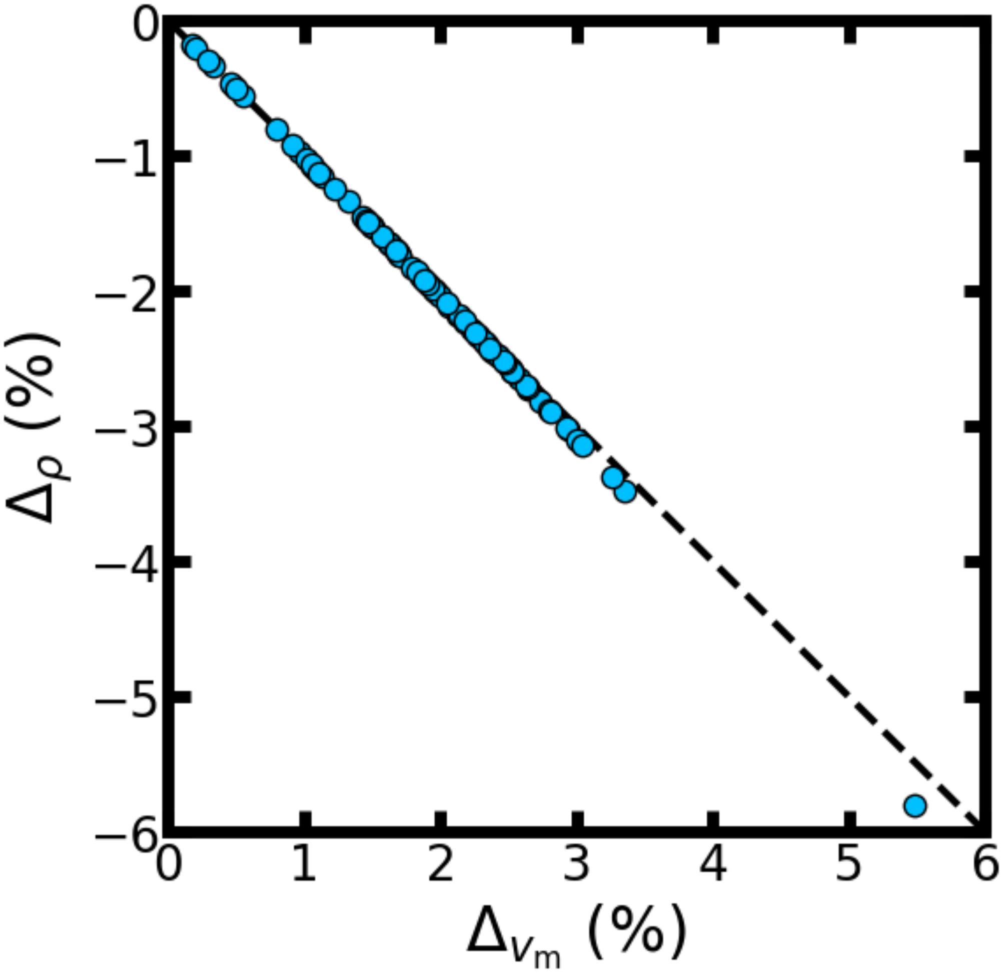

Supplementary Figure 2: Comparison of magnitude of NQEs on material density and molar volume. The change in densities, Δ_ρ, is equal to $\frac{-\Delta v_m}{1-\Delta v_m}$. At low Δv_m values, Δ_ρ and $-\Delta v_m$ are equivalent, as evident in the comparison of each value across the 92 studied systems. Most significant deviation occurs for methanal with the highest magnitude of NQEs. Source data are provided as a Source Data file.

Suppl. Note 3. Effect of Anharmonicity on Δv_m

As anharmonicity may play an important role in the magnitude of NQEs, we investigate systems with hydrogen bonding groups where their hydroxyl, amine, and thiol bond stretches are described by the anharmonic Morse function. The simulated magnitudes of NQEs are compared with the results from the original TAFFI force field.

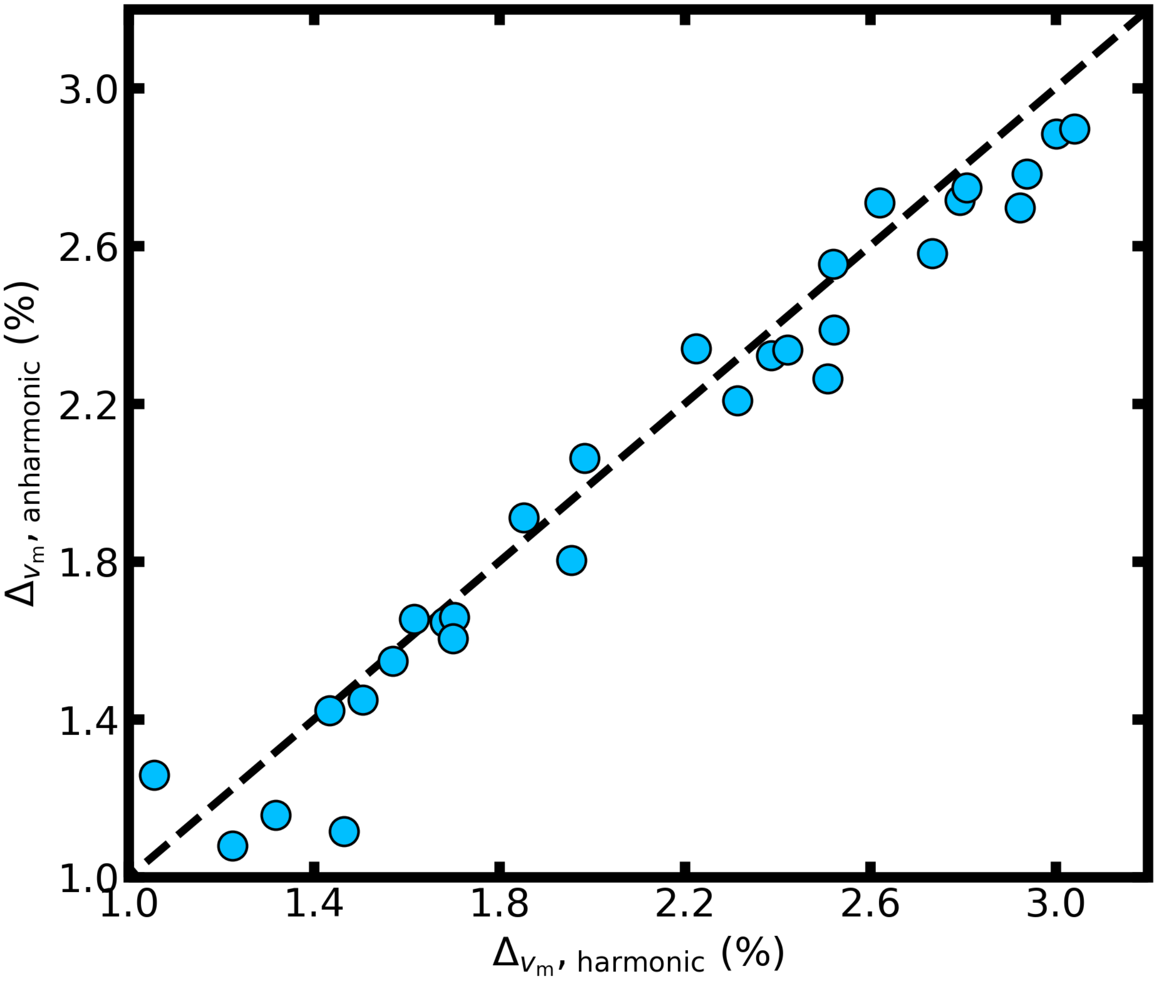

Supplementary Figure 3: Effect of bond stretch anharmonicity on NQEs. Energy functions from DFT scans of hydroxyl, amine, and thiol bond stretches at the ωB97X-D3/def2-TZVP level of theory were fitted to the anharmonic Morse potential for 29 molecules with hydrogen bonding groups. The calculated magnitude of NQEs on the molar volume are displayed against the results from the original TAFFI force field with harmonic description of the bond stretch. Source data are provided as a Source Data file.

Suppl. Note 4. Analysis NQEs Across Various Properties

While molar volume was the primary property investigated in the main text, supervised and unsupervised learning analyses were performed for other properties. The four primary descriptors (ρ, m_{w}, n_{H}, α_{P}) were used to train a random forest regressor to predict the magnitude of NQEs for each property, and SHAP analysis was performed following the procedure described in the study. The analysis was repeated using the four most correlated features using a greedy selection algorithm for each property, selected from a combination of Mordred descriptors[1] and features obtained from classical MD simulations.

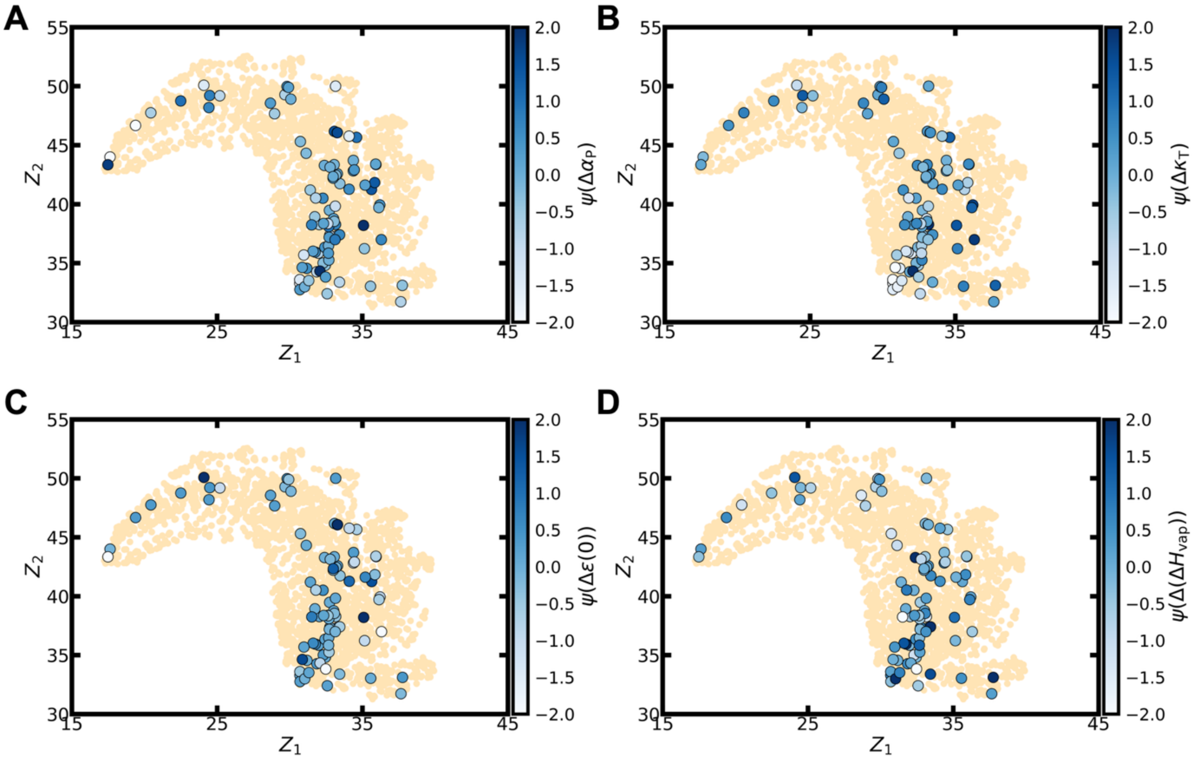

Supplementary Figure 4: Overview of magnitude of NQEs on various macroscopic properties. (A) UMAP analysis of the 92 liquid organic molecules (blue, larger markers) following the procedure outlined in the study, where the molecules are colored based on the magnitude of NQEs on (A) thermal expansion coefficients, (B) isothermal compressibilities, (C) dielectric constants, and (D) heats of vaporization. The magnitudes of NQEs for marker colors are transformed using a Yeo-Johnson power transformation, denoted by ψ. Source data are provided as a Source Data file.

Suppl. Note 5. Extended SHAP Analysis

Figure 2C in the main text has been truncated for visual clarity of the trends. We display the full observed range of data with extended x-axes below.

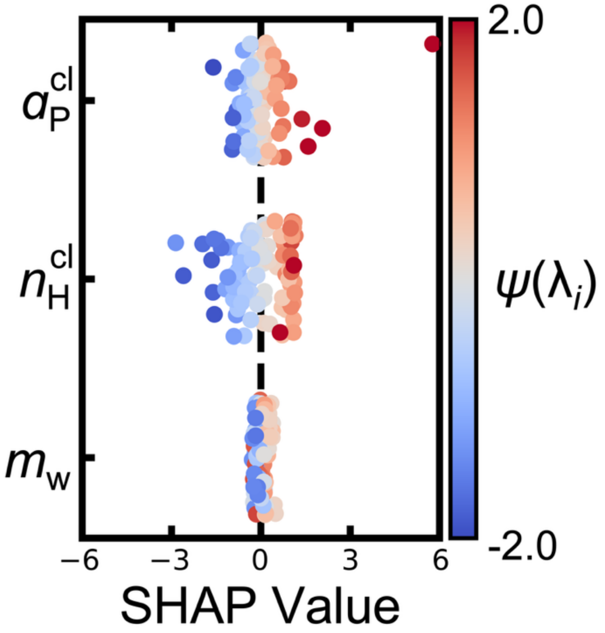

Supplementary Figure 5: Impact of feature contributions to random forest predictions based on Shapley Additive Explanations (SHAP) analysis with extended axes. The position on the x-axis indicates the impact of each feature on the model output, and the marker color indicates feature value. Feature values (λ_i) are transformed using a Yeo-Johnson power transformation, denoted by ψ. Source data are provided as a Source Data file.

Suppl. Note 6. Impact of Molecular Chemistry on Δv_m

Molecular chemistry may impact the magnitude of NQEs across a broad chemical space. To visualize this, molecules with various functional groups are categorized and the magnitude of NQEs on each system is compared.

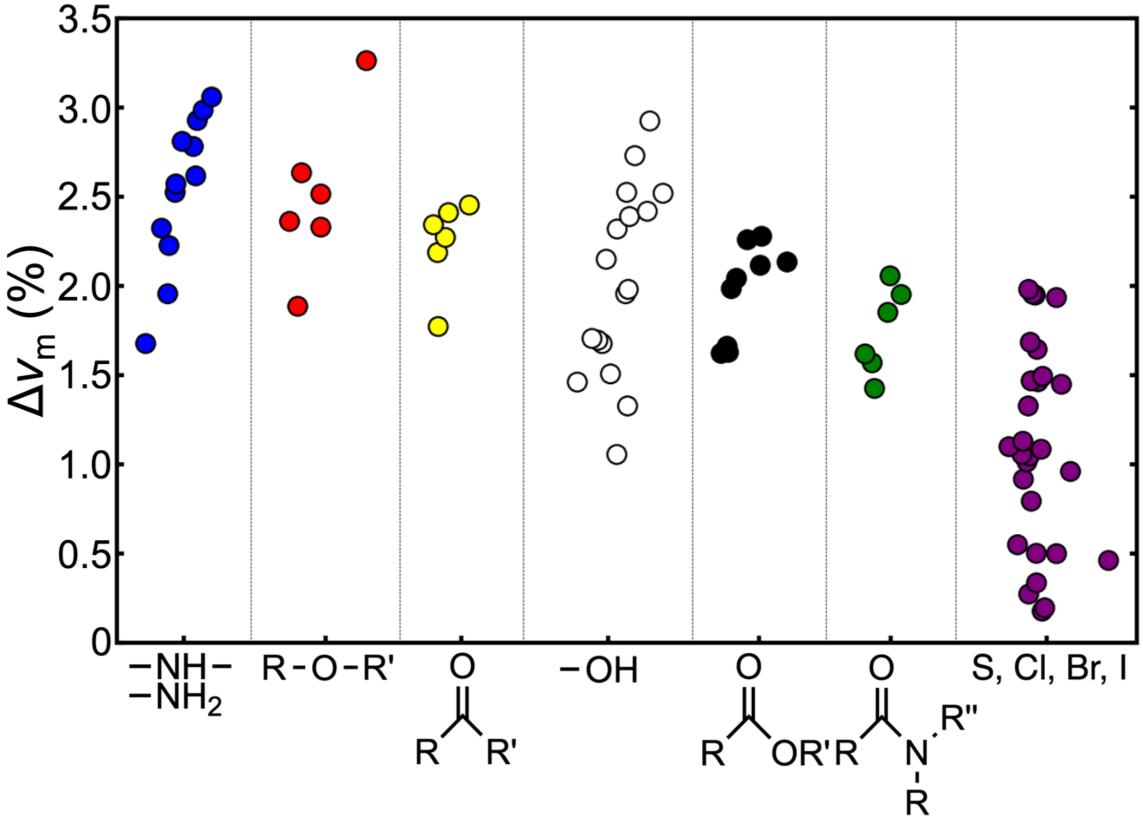

Supplementary Figure 6: Impact of specific functional groups on Δv_m. Systems containing a subset of functional groups are categorized, displaying their respective magnitudes of NQEs. Within each category, the molecules are sorted based on their σ_P values. Source data are provided as a Source Data file.

Suppl. Note 7. Distribution of T_{v_m} Across Studied Systems

Our data-driven model defines an intermediate parameter, T_{v_m}, which can be interpreted as the temperature shift required for a classical system to match the molar volume of its quantum counterpart. Below, we visualize the magnitude of these shifts across all 92 systems.

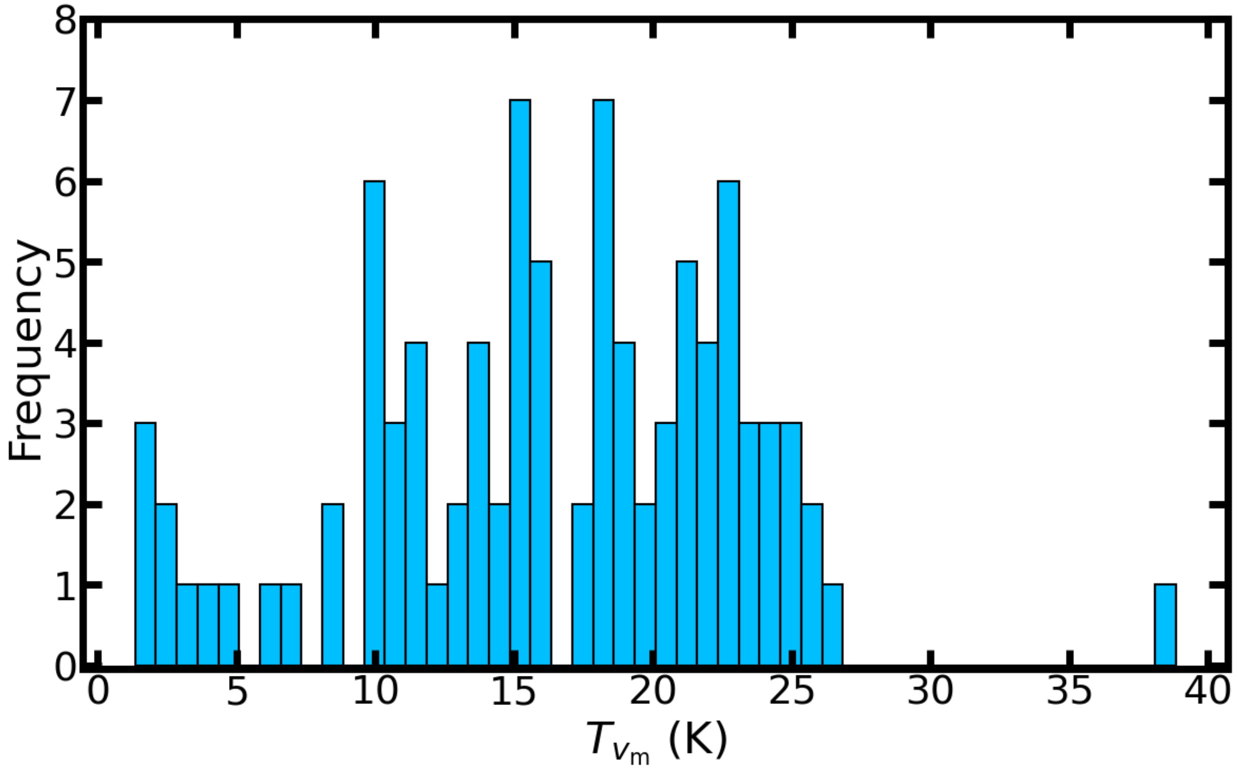

Supplementary Figure 7: Histogram of simulated T_{v_m} values for all 92 investigated molecules. The y-axis indicates the number of molecules in each corresponding T_{v_m} value on the x-axis. The data is presented over 50 bins of data within the range of T_{v_m} values. Source data are provided as a Source Data file.

Suppl. Note 8. Extended Analysis of n_H and α_P

Figure 3 in the main text has been truncated for visual clarity of the trends. We display the full observed range of data with extended x- and y-axes below.

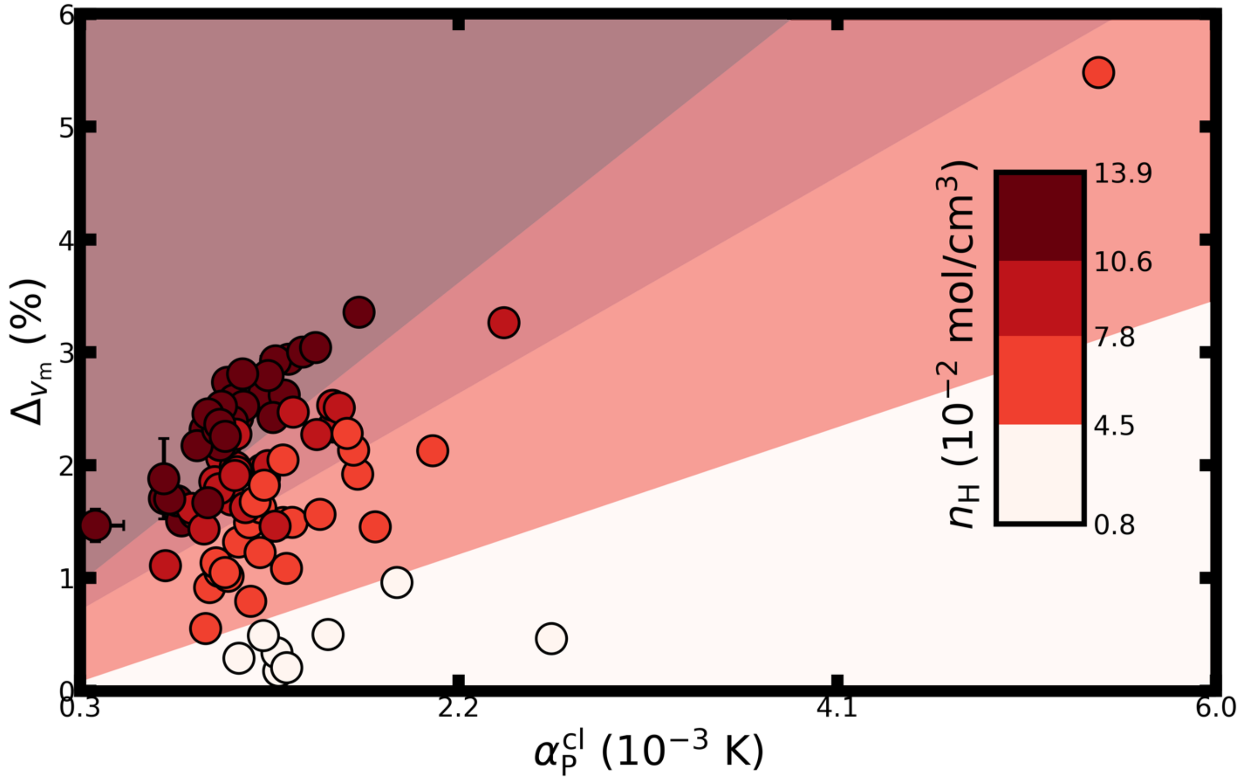

Supplementary Figure 8: Analysis of hydrogen density and thermal expansion coefficient with extended axes. Four groups of data are generated with *k*-means clustering algorithm on the respective hydrogen densities n_H; the marker colors indicate being within the range specified by the color bar. The colored regions are distinguished by support vector machine (SVM) margin lines. Error bars (black) represent the standard error of the mean obtained from four independent simulations. Source data are provided as a Source Data file.

Suppl. Note 9. Impact of Various Hydrogen-Bonding Groups on Δv_m

To evaluate the significance of hydrogen-bonding groups on the extent of NQEs, we analyze a select subset of materials with varying type and number of hydroxyl and amine groups. The four main categories include primary and secondary amines, and alcohols with one and more hydroxyl groups. The effect of the hydrogen-bonding groups on the hydrogen densities and thermal expansion coefficients are visualized below.

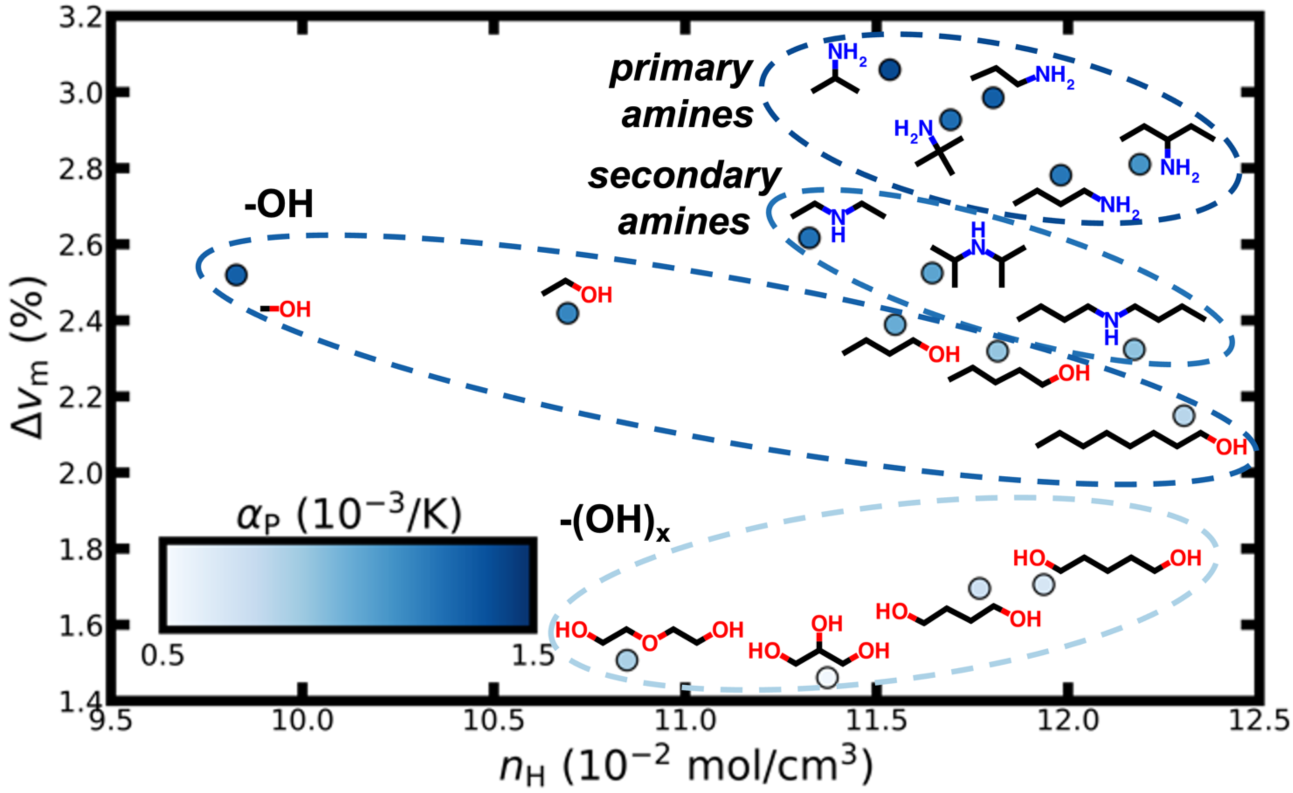

Supplementary Figure 9: Extent of nuclear quantum effects for a select group of materials containing hydrogen-bonding groups. The variation of hydrogen density and thermal expansion coefficients are displayed for four groups of materials based on their hydrogen-bonding groups: primary amines, secondary amines, single and multiple hydroxyl groups. Source data are provided as a Source Data file.

Suppl. Note 10. Effects of Molecular Branching on System Characteristics

The effects of molecular branching on the intermolecular characteristics of all systems are analyzed via pairwise comparison of branched molecules and their linear counterparts.

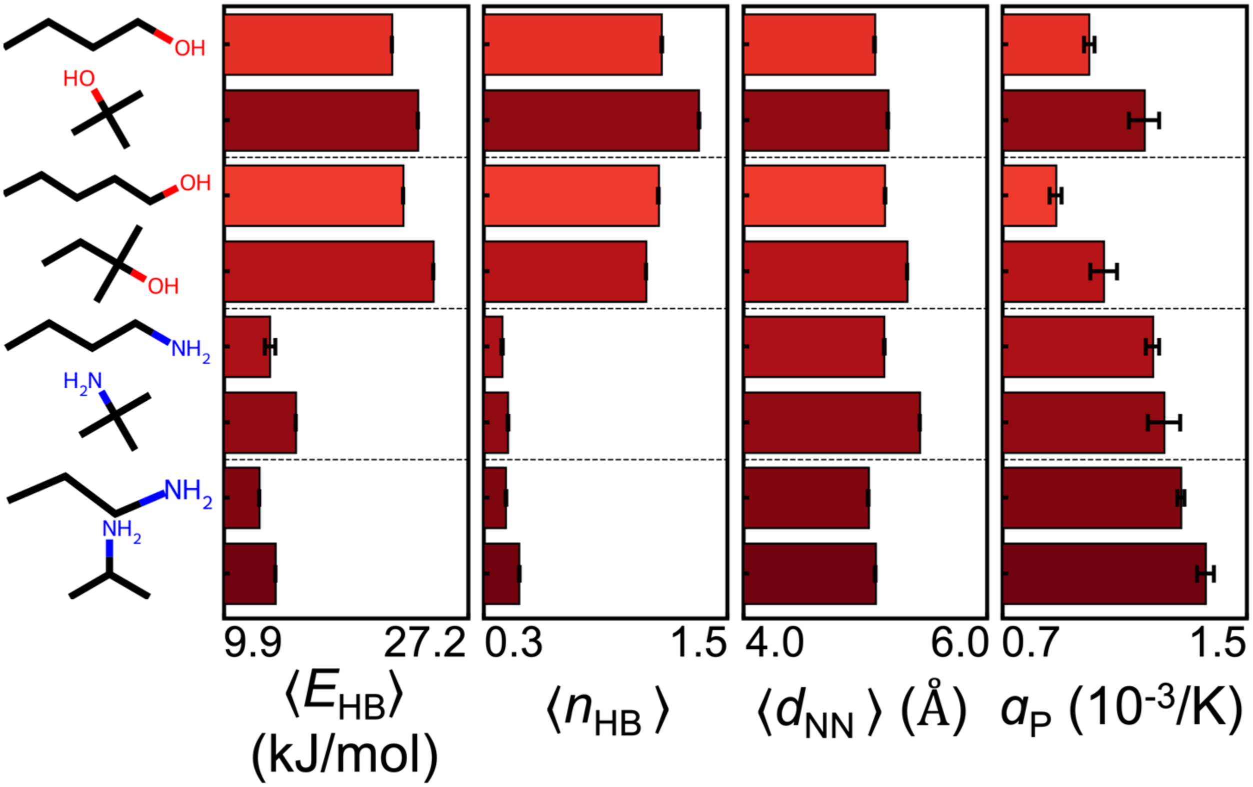

Supplementary Figure 10: Effect of molecular branching on system characteristics. The average hydrogen bond energies, hydrogen bond counts per molecule, distance to nearest neighbor, and the thermal expansion coefficients are evaluated for four pairs of branched molecules and their linear counterparts. The analyzed pairs are butan-1-ol and 2-methylpropan-2-ol, pentan-1-ol and 2-methylbutan-2-ol, butan-1-amine and 2-methylpropan-2-amine, and propan-1-amine and propan-2-amine. Darker bar colors indicate higher Δv_m. Source data are provided as a Source Data file.

Suppl. Note 11. Benchmarking of TAFFI Force Field with Experiment

Force field accuracy could impact the properties of simulated systems. Comparison of MD simulation densities and molar volumes with experimental data[2] reveals good agreement with simulation slightly overestimating for most systems. Employing PIMD simulations results in densities and molar volumes closer to experimental values.

One-tailed paired t-test was utilized to compare the accuracy of two computational methods (PIMD and classical) relative to experimental values. By analyzing the absolute errors between each method and the experimental data, we evaluated whether one approach demonstrated statistically significant improvement over the other. The test specifically examined whether the PIMD simulations produced significantly smaller errors than the classical approach. This statistical analysis provided a quantitative assessment of the relative performance of these computational techniques in reproducing experimental observations.

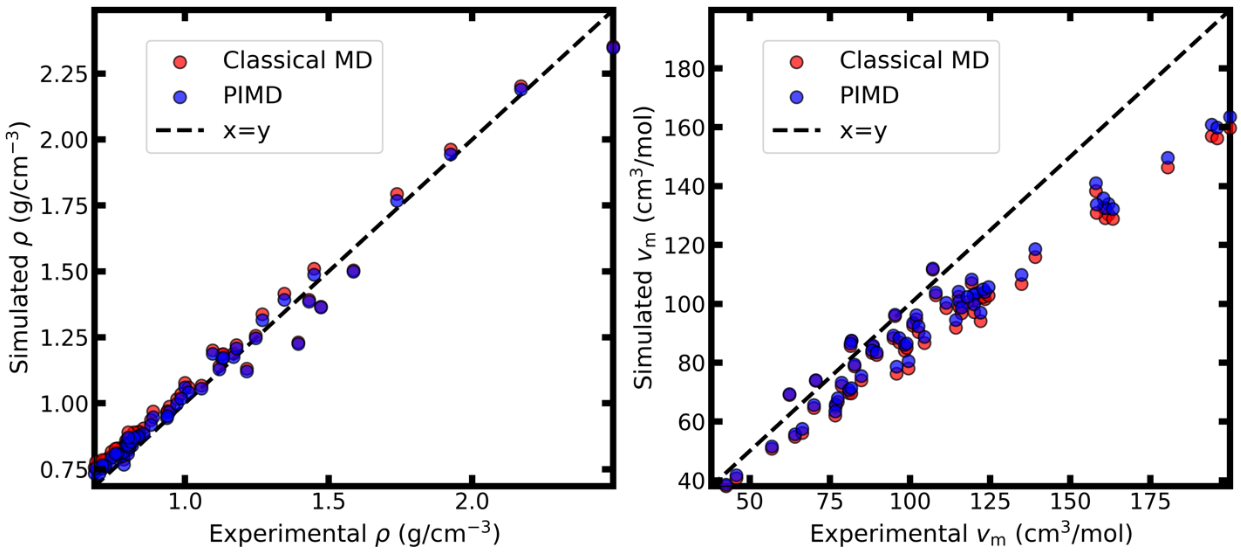

Supplementary Figure 11: Benchmarking of material densities obtained via classical (right) and PIMD (right) simulations using TAFFI force field at 298.15 K. The densities of a subset of 68 molecules with available experimental data at the ambient conditions are compared with their respective experimental values. Source data are provided as a Source Data file.

Suppl. Note 12. NVE Energy Conservation in PIMD Simulations

All simulations throughout the study use a timestep of 0.5 fs to capture the high frequency of spring elastic motion within the ring polymers. PIMD simulation of ethanol in the microcanonical ensemble is used to evaluate the conservation of total energy. The analysis displayed below includes the total energy including the spring energies of the ring-polymer and only the sum of kinetic and potential energies of the individual beads.

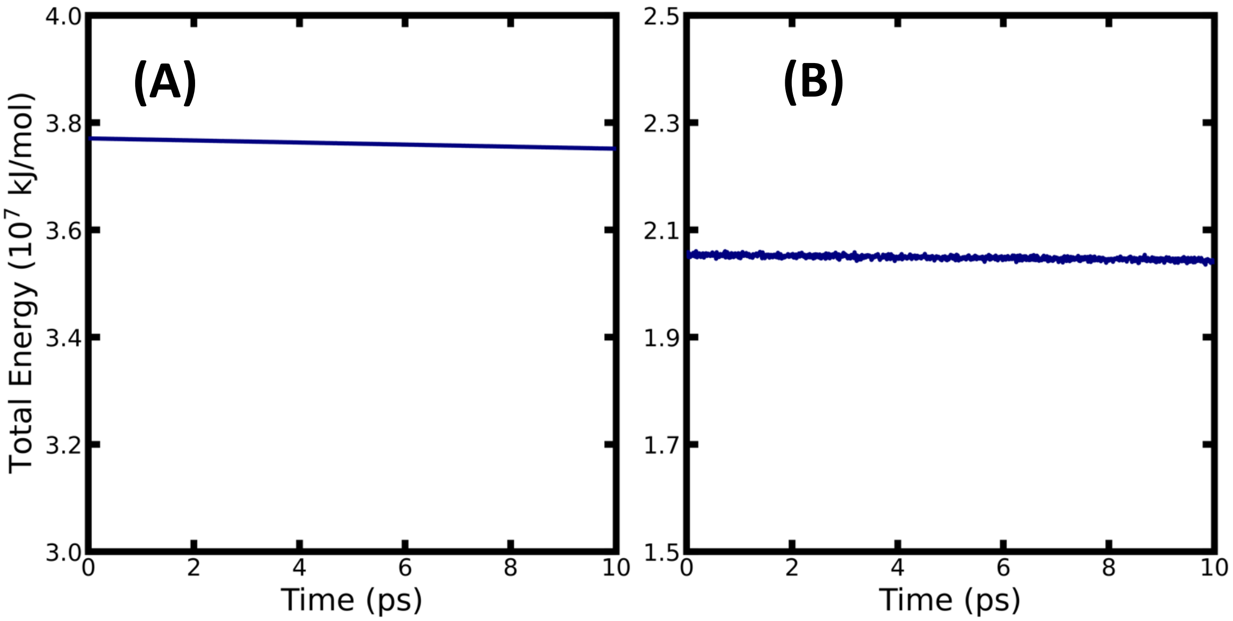

Supplementary Figure 12: Energy conservation of a PIMD simulation of ethanol in the microcanonical ensemble. The total energy is computed as the sum of kinetic and potential energies of all beads (A) including and (B) excluding the sum of potential energies of the ring-polymer springs connecting the beads. Source data are provided as a Source Data file.

Suppl. Note 13. Convergence of Density with Ring-Polymer Bead Count

To analyze whether the number of ring-polymer beads is sufficient to capture the magnitude of NQEs, PIMD simulations of propan-2-amine with bead counts of P = 1, 2, 4, 8, 16, 24, 32, and 72 were employed. Propan-2-amine is chosen for this test since it was found to exhibit some of the largest NQEs. The convergence of densities confirms that 32 beads sufficiently quantify NQEs without the additional computational requirements with higher bead counts.

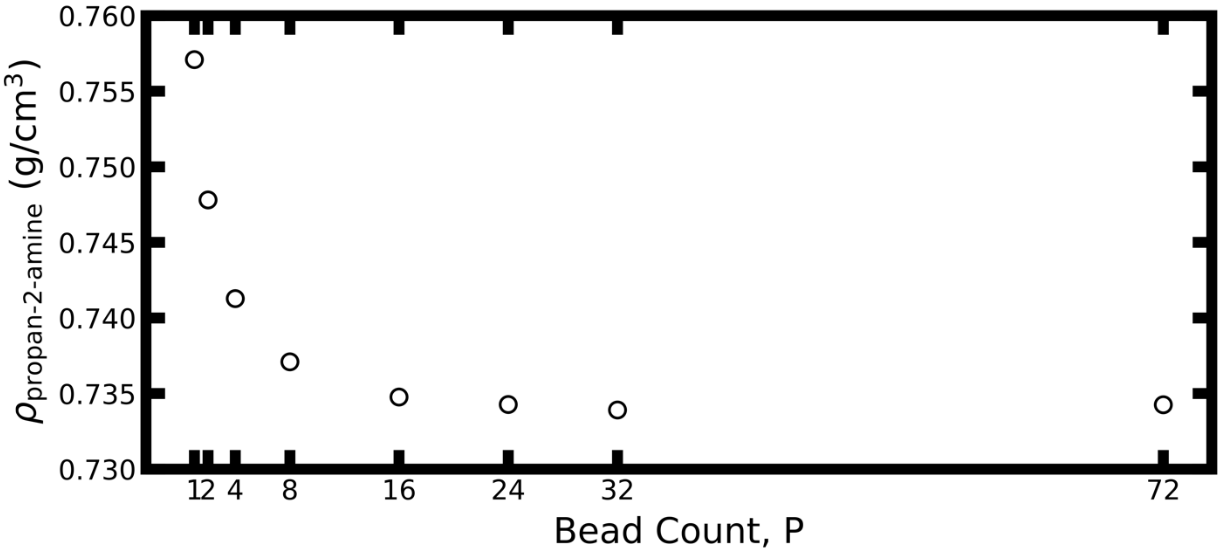

Supplementary Figure 13: Convergence of material density with increasing bead count for propan-2-amine. The densities obtained from PIMD simulations with bead counts P = 1, 2, 4, 8, 16, 24, 32, and 72 are displayed. Error bars represent the standard error of the mean from four independent simulations and are not visible under the markers. Source data are provided as a Source Data file.

Suppl. Note 14. Impact of Force Field on Δv_m

While comparison of classical and PIMD simulations provide insight on how NQEs manifest, there are computational considerations that may impact the reported findings. All simulations throughout the study employ TAFFI forcefield framework, which may impact the measured extent of NQEs. Comparison of Δv_m for a subset of 11 molecules obtained using the all-atom optimized potentials for liquid simulations (OPLS-AA) and Open Force Field (version 2.0.0 with unconstrained bonds) highlights the effect of the force field on the magnitude of NQEs. In this specific case, both force fields overestimate the extent of NQEs compared to TAFFI while the relative order is partially preserved.

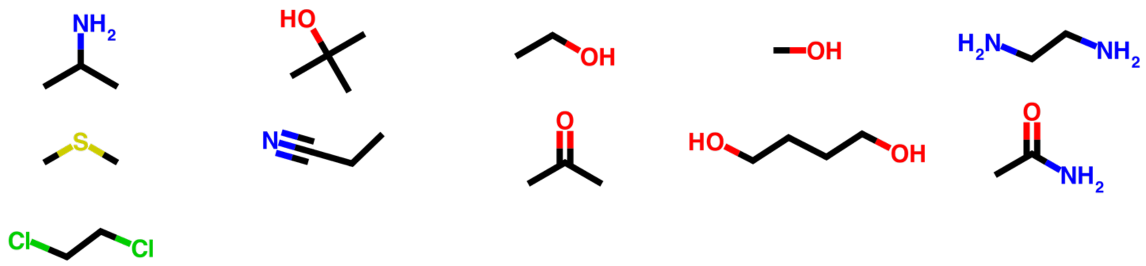

Supplementary Figure 14: List of a select subset of molecules investigated with OPLS-AA and OpenFF force fields.

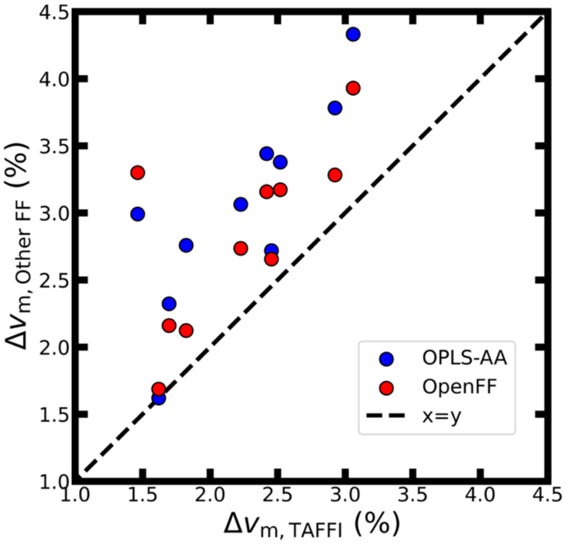

Supplementary Figure 15: Impact of force field on the computed magnitude of nuclear quantum effects for a diverse subset of molecules (displayed in Figure S3). The resulting Δv_m from classical and PIMD simulations using OPLS-AA (blue marker) and OpenFF (red marker) are displayed for each system. The black dashed line represents the x=y plot. Source data are provided as a Source Data file.

Supplementary References

1. H Moriwaki, et al., Mordred: a molecular descriptor calculator. J. Cheminform. **10**, 4 (2018).
2. C Caleman, et al., Force field benchmark of organic liquids: Density, enthalpy of vaporization, heat capacities, surface tension, isothermal compressibility, volumetric expansion coefficient, and dielectric constant. J. Chem. Theory Comput. **8**, 61–74 (2011).
